# Supplementary material for: Impact of the Rise of Artificial Intelligence in Radiology: What Do Students Think?
Source: Int J Environ Res Public Health. 2023 Jan 16;20(2):1589. doi: 10.3390/ijerph20021589 (PMC9867061; doi:10.3390/ijerph20021589)
Supplement: Supplementary file 1 [file ijerph-20-01589-s001.zip › ijerph-2144867-supplementary.pdf]

Supplementary Material- Survey

1. Would you choose Radiology as a specialty? (Q1)
   Yes
   No

2. If the previous question is affirmative, in what order of preference would you place it? (Q2)
   First
   Second
   Third
   I don't mind

3. Do you know what Artificial Intelligence (AI) is and its applications? (Q3)
   Yes
   No
   I don't know- I don't answer

4. Are you aware of how much we use AI in our daily lives? (voice and face recognition systems, Web search engines, cybersecurity, autonomous vehicles, robots, online shopping, advertising...) (Q4)
   Yes
   No
   I don't know- I don't answer

5. From what sources have you obtained information about AI? (Q5)
   Friends-Family
   Teachers-University
   Papers and Journals
   MEDIA (Television, Internet, Social networks)
   Radiologists

6. In relation to Artificial Intelligence (AI) and Radiology, check the answers you consider true. (Q6)
   a. AI is the ability of advanced computer systems to perform the same tasks as human beings (capabilities such as: reasoning, learning, creating and planning). A/True
   b. Machine learning (automatic learning) allows machines through algorithms and mathematical models to learn without being expressly programmed for it. A/ True:
   c. Deep learning: techniques based on artificial neural networks that process data and are capable of automatically recognizing patterns in biomedical images. A/ True:
   d. The use of deep learning in radiology does not require large databases of medical images for good pattern recognition. R/ False
   e. CAD (computer aided diagnosis): these are computer aided diagnosis tools developed to detect, to segment and to classify lesions or complex patterns in radiological images. A/ True

f. Radiomics: technique that consists of obtaining quantifiable information from medical images such as magnetic resonance, computed tomography or PET. They are important in detecting, evaluating and monitoring diseases. A/ True
g. Radiomics emerged from the fields of Radiology and Oncology and its application is exclusive to them. A/ False

7. Do you think that students should be trained in the use of AI? (Q7)

   Strongly disagree.

   Disagree

   Neither agree/ Nor disagree

   Agree

   Totally agree

8. Do you think AI can influence human capabilities? (No.8 quiz)
   It improves them
   It increases them
   It did not influence these capacities

9. Do you think AI can affect human autonomy by interfering with decision-making? (No.9 quiz)

   Strongly disagree.

   Disagree

   Neither agree/ Nor disagree

   Agree

   Totally agree

10. Do you think AI should follow ethical principles? (Q10)
    Strongly disagree.

    Disagree

    Neither agree/ Nor disagree

    Agree

    Totally agree

11. What would you consider to be the biggest drawback of using AI in medicine? (Q11)
    They cannot interpret the patient in a global clinical context:
    High cost of its implementation
    Possible vulnerability of the right to privacy of patients
    Necessary training in the management of AI for professionals

12. What role would you assign to the use of AI in radiology? (Q12)
    Support Role

Preponderant
I will not use it

13. Do you think the rise of AI could replace radiologists? (Q13)
    Strongly disagree.
    Disagree
    Neither agree/ Nor disagree
    Agree
    Totally agree

14. Will the way radiologists work change with the impact of AI? (Q14)
    Strongly disagree.
    Disagree
    Neither agree/ Nor disagree
    Agree
    Totally agree

15. What do you think should be the role of radiologists? (Q15)
    Lead the algorithm validation process, contribute their experience in the global clinical approach of patients and make the final decision
    Your work should continue as usual and not use AI because it is unreliable
    They should be relegated to the background since the implementation of AI will be the future

16. In what aspects do you think the use of AI improves in the radiology service? (Q16)
    It favors early diagnosis and treatment of diseases
    It improves the management and quality of radiology services
    It allows radiologists to focus on patient care in a general clinical context by making their work easier
    It reduces the number and qualification of the professionals needed in the service

17. Taking into consideration the impact that AI can have on medical specialties, would your preferences change when choosing? (Q17)
    Yes
    No
    Maybe
